# Supplementary material for: Excitonic signatures of ferroelectric order in parallel-stacked MoS2
Source: Nat Commun. 2024 Aug 31;15:7595. doi: 10.1038/s41467-024-52011-3 (PMC11366029; doi:10.1038/s41467-024-52011-3)
Supplement: Supplementary file 1 — Supplementary Information [file 41467_2024_52011_MOESM1_ESM.pdf]

Excitonic signatures of ferroelectric order in parallel stacked MoS_2

Swarup Deb*[1], Johannes Krause[1], Paulo E. Faria Junior[4], Michael Andreas Kempf[1], Rico Schwartz[1], Kenji Watanabe[2], Takashi Taniguchi[3], Jaroslav Fabian[4], and Tobias Korn**[1]

[1]Institute of Physics, University of Rostock, Albert-Einstein-Str. 23, Rostock 18059, Germany
[2]Research Center for Electronic and Optical Materials, NIMS, 1-1 Namiki, Tsukuba 305-0044, Japan
[3]Research Center for Materials Nanoarchitectonics, NIMS, 1-1 Namiki, Tsukuba 305-0044, Japan
[4]Institute for Theoretical Physics, University of Regensburg, 93040 Regensburg, Germany
*swarupdeb2580@gmail.com
**tobias.korn@uni-rostock.de

Supplementary Information

S1. Device fabrication

*h*BN flakes of various thicknesses were mechanically exfoliated onto a Si/SiO_2 substrate which were annealed on a hot-plate at 360°Cto clean up scoth tape residue. 3R-MoS_2, obtained from HQ Graphene, were exfoliated onto polydimethylsiloxane (PDMS). MoS_2 flakes were selected according to their optical contrast. Chosen TMD flakes were stamped on the *h*BN surface directly from PDMS at ambient conditions. We use a polycarbonate (PC)/PDMS-based hot pickup method[1] to transfer *h*BN flakes from bare Si/SiO_2 substrate to encapsulate *h*BN/TMD stack.

A few layer thick graphite flakes (procured from NGS Naturgraphit), exfoliated on PDMS, are deterministically stamped to make external contact to the sample. Subsequently we wire bond the graphite flake using two component conductive silver epoxy. Keithley 2401 source meter were employed to apply external gate voltage.

S2. KPFM measurements

KPFM measurements were performed using Park System NX20 AFM either in tapping or in non-contact scanning mode. The electrostatic signal was measured at side-band frequencies using two built-in lock-in amplifiers. We used PointProbe Plus Electrostatic Force Microscopy (PPP-EFM) n-doped tips with a conductive coating acquired from Nano and More GMBH. The mechanical resonance frequency of the tips was ∼75 kHz, and the force constant was 3 N/m. The cantilever was excited with an AC voltage to perform KPFM measurements, with an amplitude of 1.5-2.5 V and a frequency of 2-4 kHz. In the closed-loop measurements, the DC voltage was controlled by a bias servo to obtain the surface potential.

For unencapsulated sample (Sample 1 c.f. Fig. 1) we used non-contact mode of operation. The average height above the surface was controlled via a two-pass measurement. The first pass records the topography, whereas, in the second pass, the tip follows the same scan line with a predefined lift (typically 4-5 nm) and measures the KPFM signal. In case of Sample 4 c.f. Fig. 3 where we used a ∼85 nm *h*BN for top encapsulation, we operated the AFM in tapping mode and the KPFM measurements were done in a single pass. Images were acquired using the Park SmartScan software, and the data was analyzed with the Gwyddion software.

S3. Reflectance measurements and spatial filtering

All optical measurements were performed in flow-type cryostats with an accessible temperature range of 4K to room temperature. The samples were kept under vacuum for all the optical measurements. All the experiments were performed in back-scattering geometry.

For white light reflectance measurements, the samples were illuminated with a quartz tungsten halogen lamp. The collimated beam was focused on the sample using an 80X objective. The reflected light from the sample was collected using the same objective and spectrally resolved using a spectrometer and a charge-coupled detector. To obtain the hyperspectral map of the samples, the cryostat with the samples inside, was moved with respect to the fixed optical spot using a computer-controlled XY stage. The data was analyzed with LabView and Origin software.

In the detection path, before the spectrograph, we introduce a home-built *Spatial-Filtering* module to enhance the spatial resolution. The input side of the spatial filter consists of an aspheric lens mounted on a Z-translator. It focuses the reflected beam onto a pinhole. The pinhole was carefully aligned to the beam path with the help of an XY translator. A fraction of the focused light passes through the pinhole aperture. Another aspheric lens was used to collect the beam from the pinhole and collimate along the detection path.

S4. Time resolved reflectivity and Kerr ellipticity

In the pump-probe setup, two separately tunable pulsed lasers (Toptica: femtoFiberPro) were used, one for the pump and the other for the probe laser beam. Each system emitted with a pulse repetition rate of 80 MHz, a spectral width of 6 meV, a pulse duration of about ∼200 fs. Probe and pump pulses were electronically time-synchronized and amplitude-modulated with different chopping frequencies, adding up to a sum modulation frequency of ∼950 Hz. A mechanical delay line changed the path length of both beams and, thus, the time offset of both pulses. Through an achromatic $\lambda/4$ plate, the pump beam was circularly polarized to either left- or right-handed helicity. After reflection the pump beam was filtered out by a long-pass filter while the polarization and signal amplitude of the probe beam were measured. The polarization of the probe beam was analyzed for its ellipticity by a combination of a $\lambda/4$ plate, a Wollaston prism, and two photodiodes (ThorLabs PDB210A Si Photodetector). The difference signal of the two diodes was fed into a lock-in amplifier, yielding a TRKE signal. The sum signal of the diodes was obtained by using an external adder and fed into a second lock-in amplifier, yielding the TRΔR signal. Both lock-in amplifiers were fed the same reference frequency given by the modulation sum frequency. With this approach, it was possible to measure the TRKE and TRΔR at the same time, cutting the needed measurement time in half, compared to, as commonly performed, subsequent measurements. The TRKE setup is described in detail elsewhere.[2,3]

S5. Density functional theory

To investigate the electronic properties and optical selection rules of the five-layer (5L) MoS_2 with 3R stacking, we performed density functional theory (DFT) calculations using the all-electron full-potential linearized augmented plane-wave (LAPW) method implemented in the Wien2k code.[4] We consider the Perdew-Burke-Ernzerhof[5] exchange-correlation functional with van der Waals interactions included via the D3 correction.[6] The wave function expansion into atomic spheres uses orbital quantum numbers up to 10 and the plane-wave cut-off multiplied with the smallest atomic radii is set to 8. Spin–orbit coupling was included fully relativistically for core electrons, while valence electrons were treated within a second-variational procedure[7] with the scalar-relativistic wave functions calculated in an energy window up to 2 Ry. Self-consistency was achieved using a two-dimensional Monkhorst–Pack k-grid with 15×15×1 points and a convergence criteria of 10^{-6} e for the charge and 10^{-6} Ry for the energy. We used the structural parameters taken from Ref. 8, i. e., the in-plane lattice parameter is 3.191 Å, the thickness of a single MoS_2 layer is 3.116 Å, the interlayer distance between adjacent MoS_2 layers is 3.094 Å, and a vacuum region of 20 Å was used to avoid interaction between

the heterostructures' replicas. We explored all the possible combinations of 3R stackings in a 5L system leading to a total of 10 different structures, shown in Fig. S18 (see also Fig. S12). The total energy of these different systems are nearly the same, as shown in Fig. S19, supporting the appearance of different domains in the studied samples. The calculated band structures with spin-orbit coupling along the $\Gamma - K - M$ line are shown in Fig. S20 with the color-code representing the spin expectation value in the out-of-plane direction. A zoom of the conduction and valence bands in the vicinity of the K point is shown in Fig. S21, revealing that the energy bands that generate A and B excitons show strong spin-valley locking. The wave functions presented in Fig. 2 of the main text were evaluated without spin-orbit coupling. Turning to the selection rules at the K point, we present in Fig. S22 the absolute value of the dipole matrix elements ($|p_{cv}| = \frac{\hbar}{m_0} |\langle c, K| \vec{p} \cdot \hat{\alpha} | v, K \rangle|$ calculated within the LAPW basis set,[9] with $\hat{\alpha} = s^+, s^-, z$ encoding the light polarization) as a function of the band labels. In Fig. S23 we display the oscillator strength, i. e., $|p_{cv}|^2$, that enters the absorption spectra. In Figs. S24 and S25 we present $|p_{cv}|$ and $|p_{cv}|^2$ as a function of the transition energy only for s^+ polarization (the dominant contribution due to the stron spin-valley locking). Our results reveal that variation of the transition energies is on the order of 10–20 meV for the different stackings.

S6. Exciton binding energies

To evaluate the effect of the asymmetric dielectric surroundings of the 5L MoS_2 systems, we calculated the binding energies for the intralayer A excitons located at each layer. We employed the effective Bethe-Salpeter equation[10,11] formalism, assuming non-interacting parabolic bands for electrons and holes.[12,13] We consider the average value of effective masses taken from the DFT calculations ($m_v = 0.515$ and $m_c = 0.415$) and the electrostatic potential for each layer is obtained numerically by solving the Poisson equation in k-space, assuming the different regions to have dielectric constants of $\varepsilon(MoS_2) = 15.45$ (from Ref. 14), $\varepsilon(\text{hBN}) = 4.5$ (from Ref. 15), and $\varepsilon(\text{air}) = 1$. We consider each MoS_2 layer to have an effective thickness of 6.232 Å, taken as twice the value of the physical thickness of the TMDC.[16] The calculated dielectric constants experienced by the intralayer excitons at each MoS_2 layer are show in Fig. S26. The potential at each layer, l, takes the form $V(\vec{k}) \sim [k\epsilon_l(k)]^{-1}$, with $l = 1, \ldots, 5$. The resulting exciton binding energies are given in Table S1, showing variations of up to 30 meV, a similar energy scale of the optical transitions discussed in Figs. S24 and S25. For the structures with mixed layer contributions (MX-XM-MX-XM, XM-MX-XM-MX, and XM-XM-MX-MX) we average the energy transitions and leave the intralayer exciton potentials intact. Since these quantities (transition energies and exciton binding energies) operate on the similar energy scales, our conclusions remain valid. Thus, combining the DFT transition energies and dielectric effects within the exciton picture, we present the resulting absorption exciton spectra in Fig. S27. We used the same value of the dipole matrix element for all transitions. DFT underestimates the band gap and therefore our calculations are red-shifted by ∼480 meV in comparison with the experimental values. Nonetheless, the exciton absorption peaks are 10–30 meV apart depending on the particular stacking (ferroelectric domains), in excellent agreement with the experimental findings shown in Fig. 1d of the main text.

Table S1 | Calculated intralayer exciton binding energies in meV.

L1	L2	L3	L4	L5
−131.6	−117.5	−116.6	−124.2	−152.2

S7. Extended data

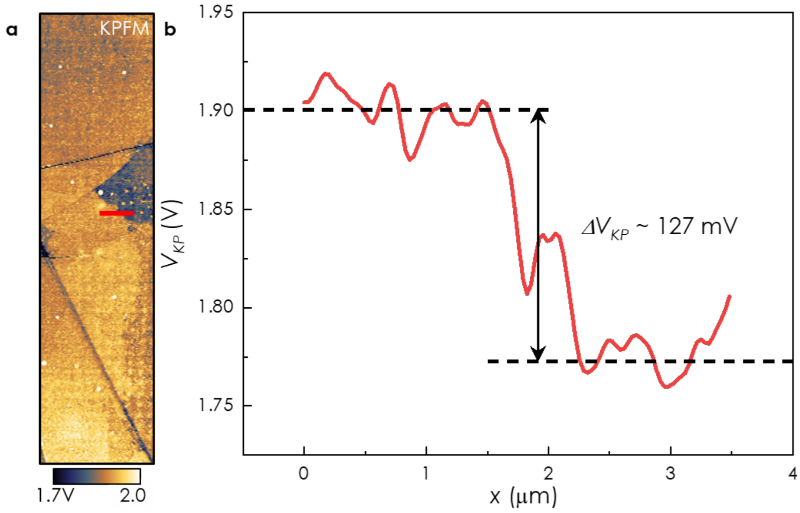

Figure S1 | Multi-polarization states in naturally grown 3R-MoS$_2$. a. Surface potential map copied from Fig.1b of the main text for reference. **b.** Typical line cut of the lateral surface potential profile across a domain wall separating regions I and II, marked by the red line in panel a. The measured potential variation, $\Delta\ V_{KP} \sim 127\,\text{mV}$ agrees well with earlier reported values in this material.[17,18]

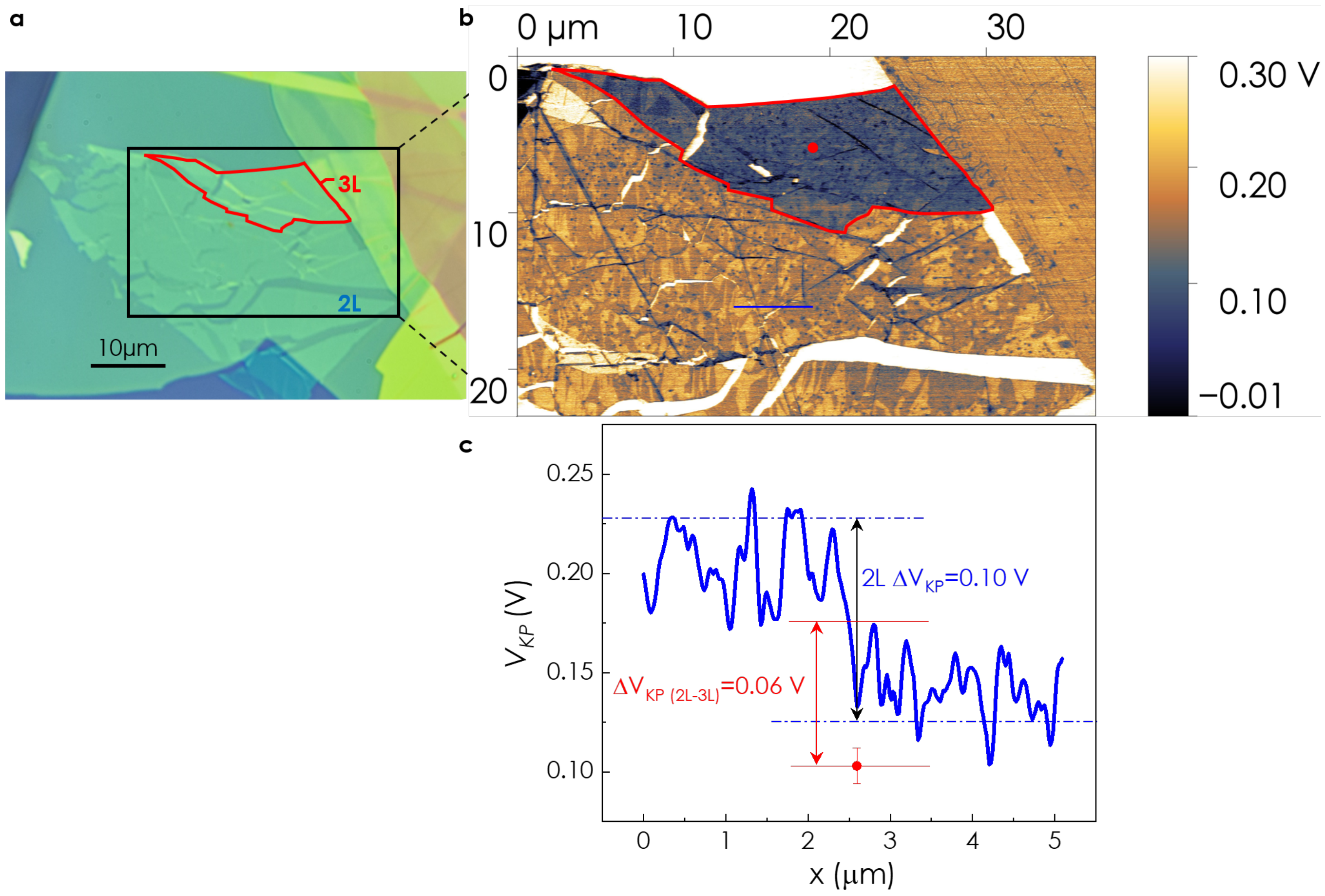

Figure S2 | Inducing ferroelectric domains in naturally grown 3R-MoS$_2$. a. Optical micrograph of a bi- and trilayer 3R-MoS$_2$ flake stamped on Si/SiO$_2$/hBN with deliberate shear perturbation (horizontal force) during the transfer from PDMS. **b.** Surface potential map within the area enclosed by the black rectangle in a. Densely packed ferroelectric domains can be identified by the difference in contrast. **c.** A typical line cut of the lateral surface potential profile across domains in the bilayer region, marked by the blue line in panel b. The red circle is the surface potential from the trilayer region.

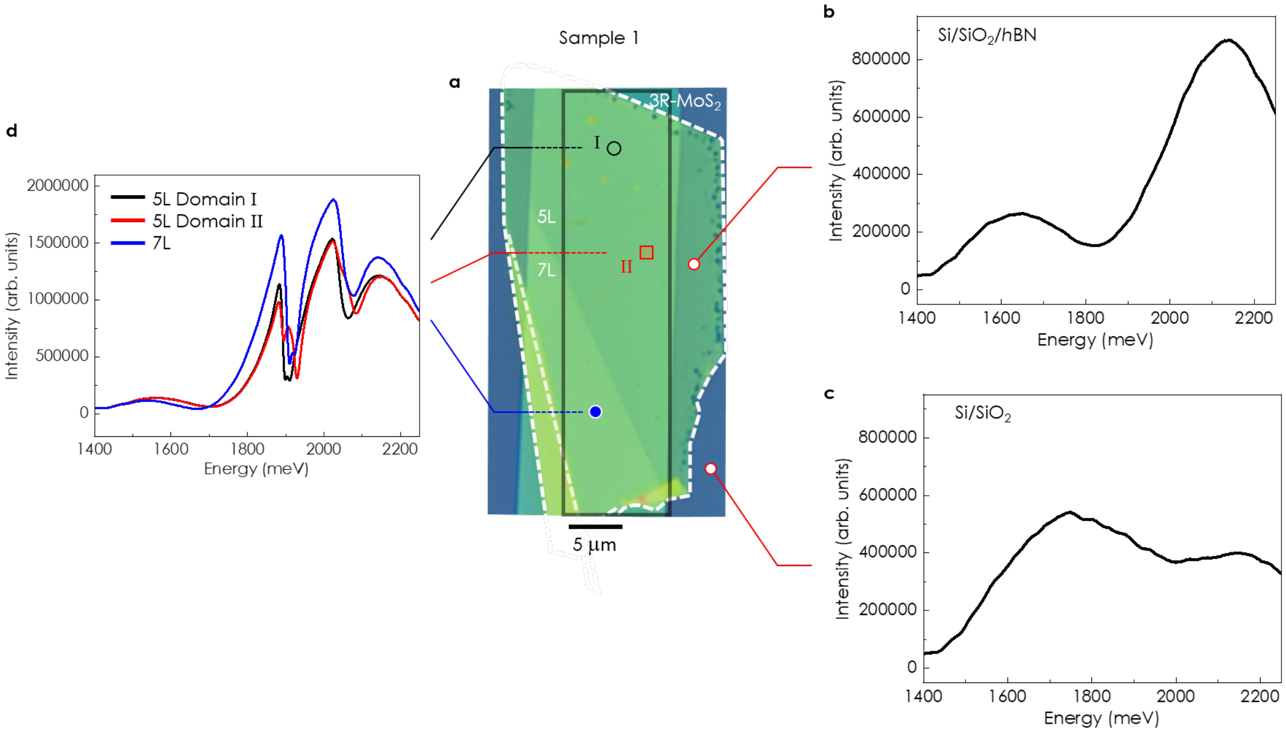

Figure S3 | Reflectance spectra. a. Optical micrograph of Sample 1. **b-d.** As recorded reflectance spectra at 4K from various spatial locations, as indicated by symbols and lines.

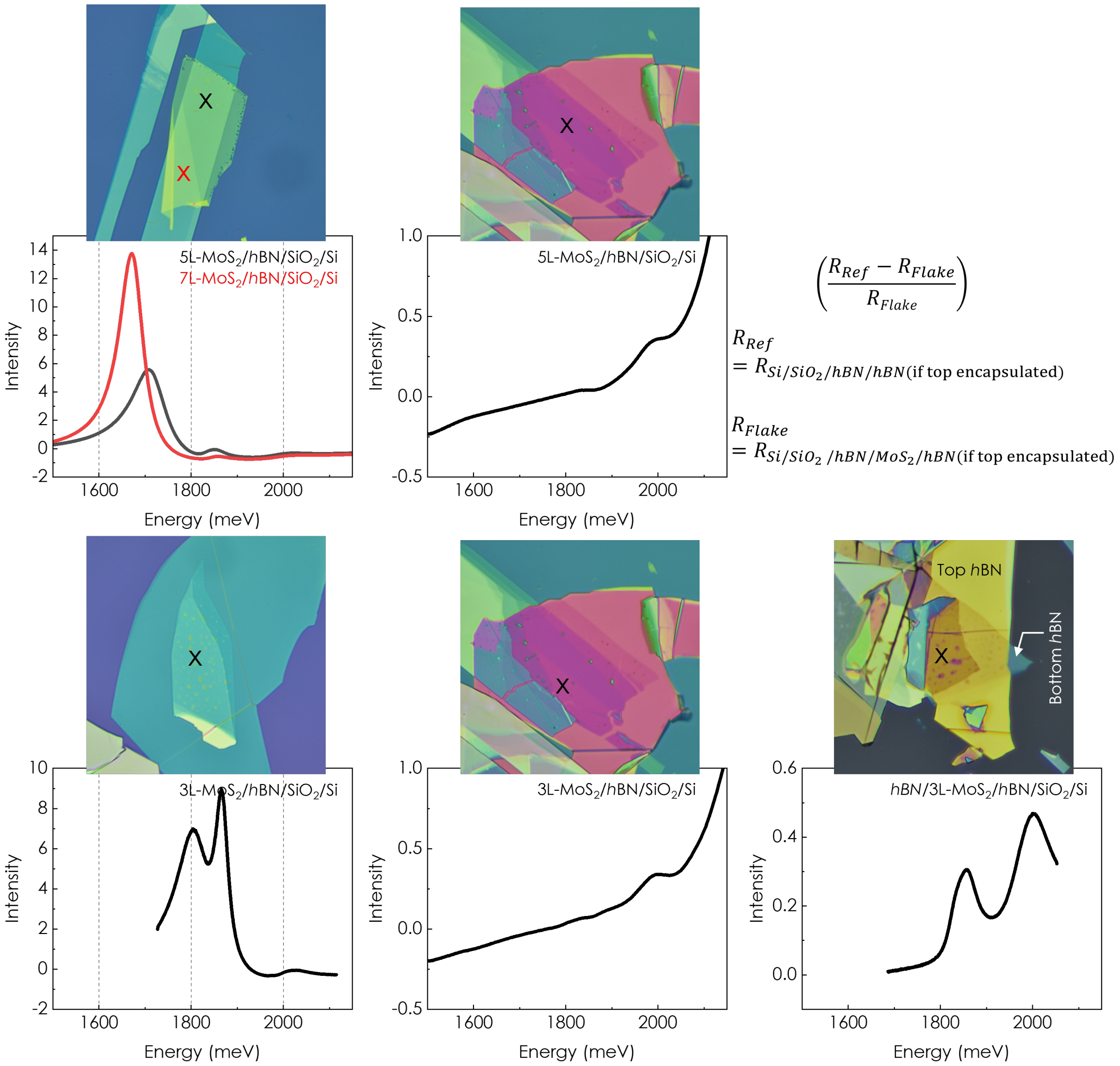

Figure S4 | Cavity effect on reflection contrast intensity. Room temperature reflectance contrast spectra of four different samples along with their optical microscopic images. Samples with a turquoise appearance (left panels) shows a pronounced Γ-Q or K-Q and X_A peaks. Conversely, sample prepared on thicker hBN, appearing pink (central panels), exhibit no detectable ΔR/R intensity in the lower energy range, and the X_A transition is only weakly visible. However, the X_B feature is relatively stronger in this sample. In the case of an orange-colored sample (top-encapsulated, right panel), X_A and X_B transitions are prominently present, while the lower energy feature remains invisible. Here we have used RC = $\Delta R/R_{Flake}$.

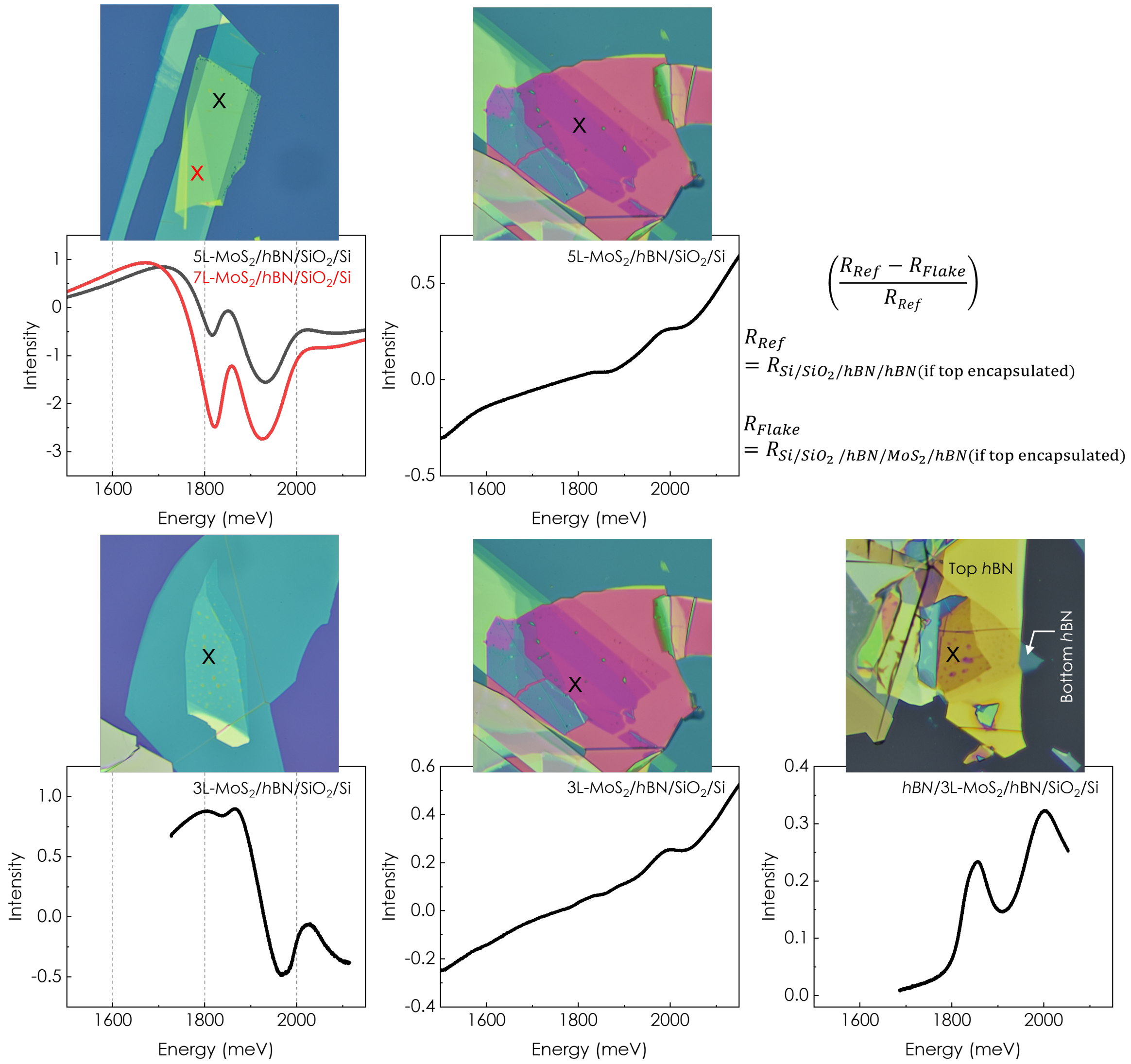

Figure S5 | Cavity effect on reflection contrast intensity. The same as in Fig. S4 with the exception of RC being equal to $\Delta R/R_{Ref}$.

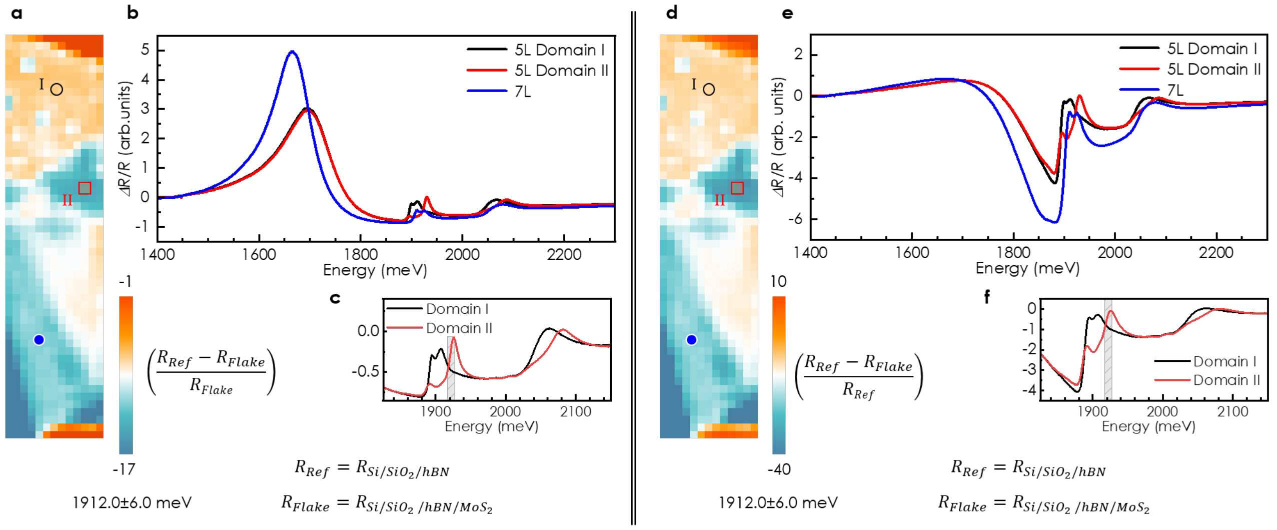

Figure S6 | Comparison of $(\mathbf{R_{Ref} - R_{Flake}})/\mathbf{R_{Flake}}$ with $(\mathbf{R_{Ref} - R_{Flake}})/\mathbf{R_{Ref}}$ for Sample 1. Integrated intensity map of $\Delta R/R$ at the X_A spectral region, i.e., from 1906 to 1918 meV along with low-temperature reflectance contrast spectra from various spatial locations marked by symbols of corresponding color in the map (black - 5L domain I, red 5L domain II, and blue - 7L). We have used in **(a-c.)** RC = $(R_{Ref} - R_{Flake})/R_{Flake}$ (as in Fig. 1c and d) and in **(d-f.)** RC = $(R_{Ref} - R_{Flake})/R_{Ref}$.

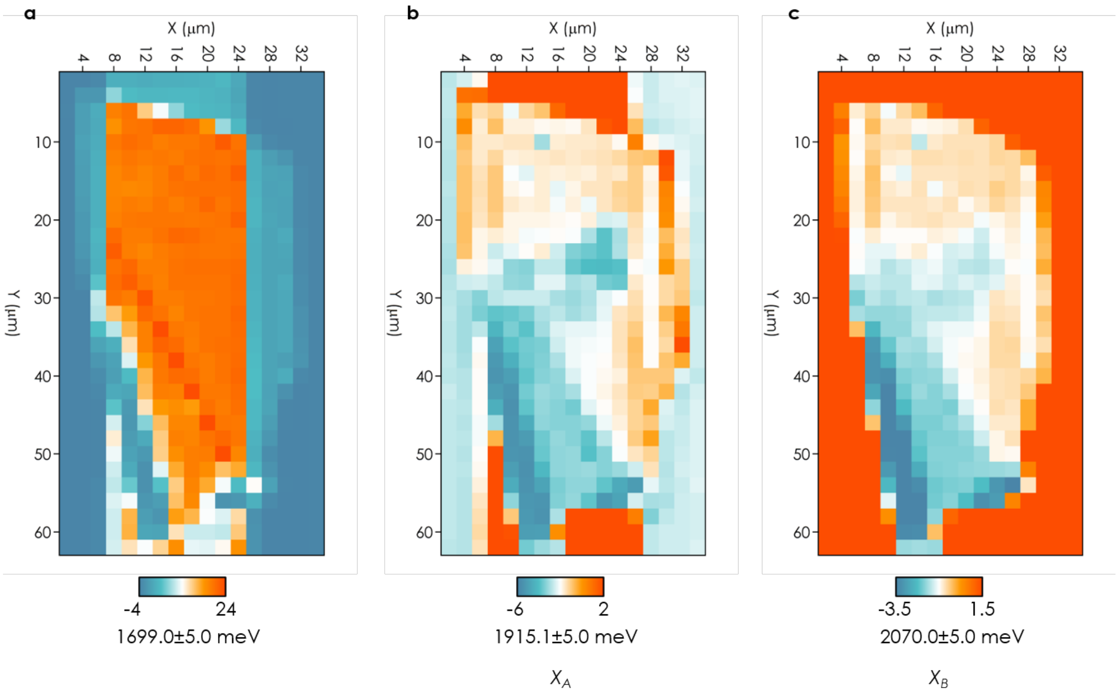

Figure S7 | Reflection contrast intensity of ferroelectric domains in few-layer 3R-$\mathbf{MoS_2}$. a-c. Low-temperature integrated intensity map of ΔR/R of the complete flake at the Γ-Q or K-Q peak, X_A, and X_B spectral region, respectively.

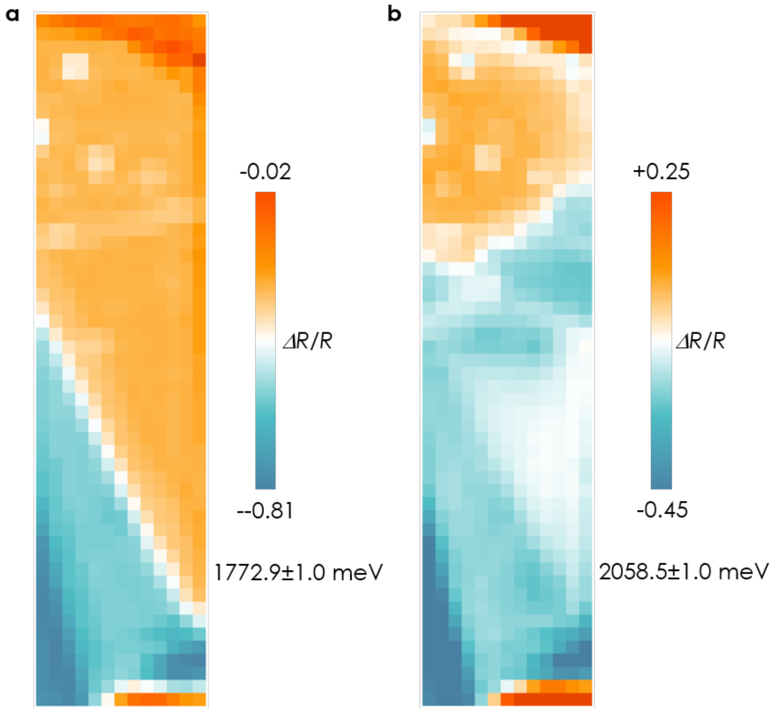

Figure S8 | High resolution reflection contrast intensity of ferroelectric domains in few-layer 3R-MoS_2. a-b. Low-temperature integrated intensity map of ΔR/R of the selected region of flake as Fig. 1 in the main text at the low energy tail and B_x spectral region, respectively. This data set differs from Fig. S7 in terms of its spatial and spectral resolution.

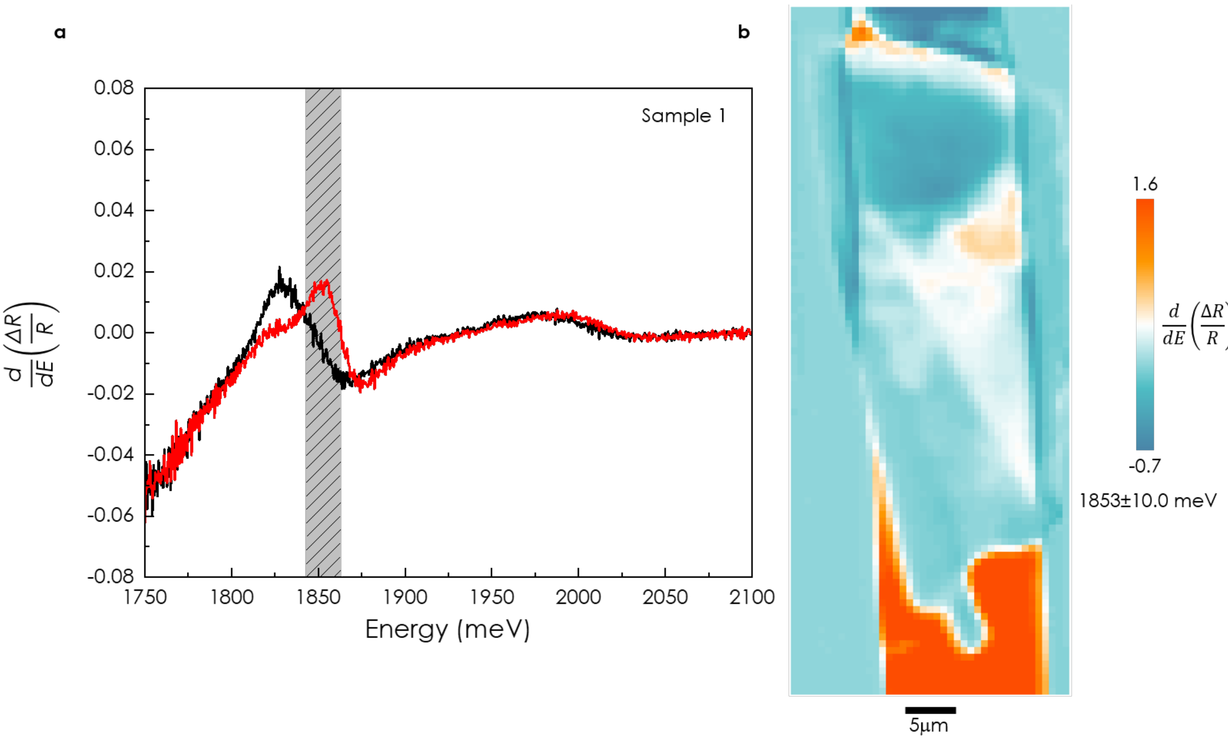

Figure S9 | First derivative of reflection contrast at room temperature. a. Numerically calculated first derivative of reflectance contrast spectra from Domain I and II. **b.** Sum intensity map of $\frac{d}{dE}\left(\frac{\Delta R}{R}\right)$ at the X_A spectral region i.e. from 1843 to 1863 meV (gray bar in a). The analysis highlights that the ferroelectric domains can be probed at room temperature, opening up an unprecedented opportunity for potential applications.

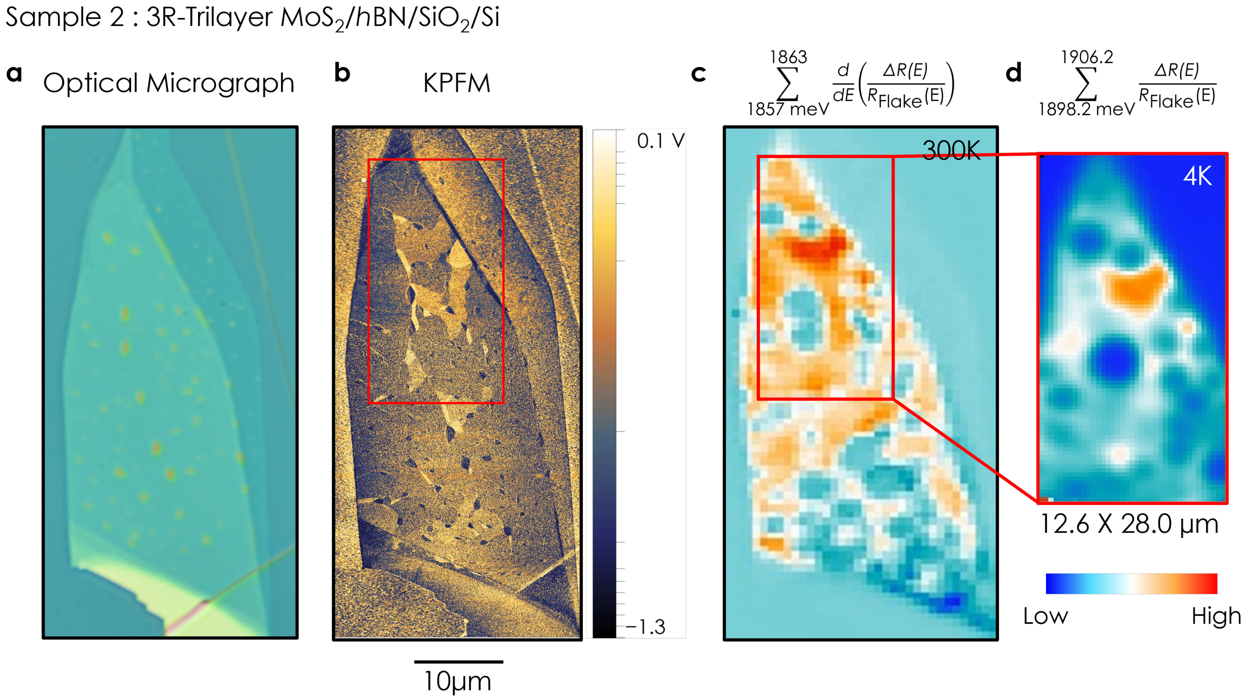

Figure S10 | Reflection contrast imaging of ferroelectric domains in trilayer 3R-MoS$_2$. a. Optical micrograph of a 3R-MoS$_2$ flake on Si/SiO$_2$/hBN. **b.** Surface potential map of the entire flake. **c.** Room temperature sum intensity map of $\frac{d}{dE}\left(\frac{\Delta R}{R}\right)$ at the X_A spectral region i.e. from 1857 to 1863 meV. **d.** Low-temperature reflectance contrast map at the X_A spectral region i.e. from 1898.2 to 1906.2 meV.

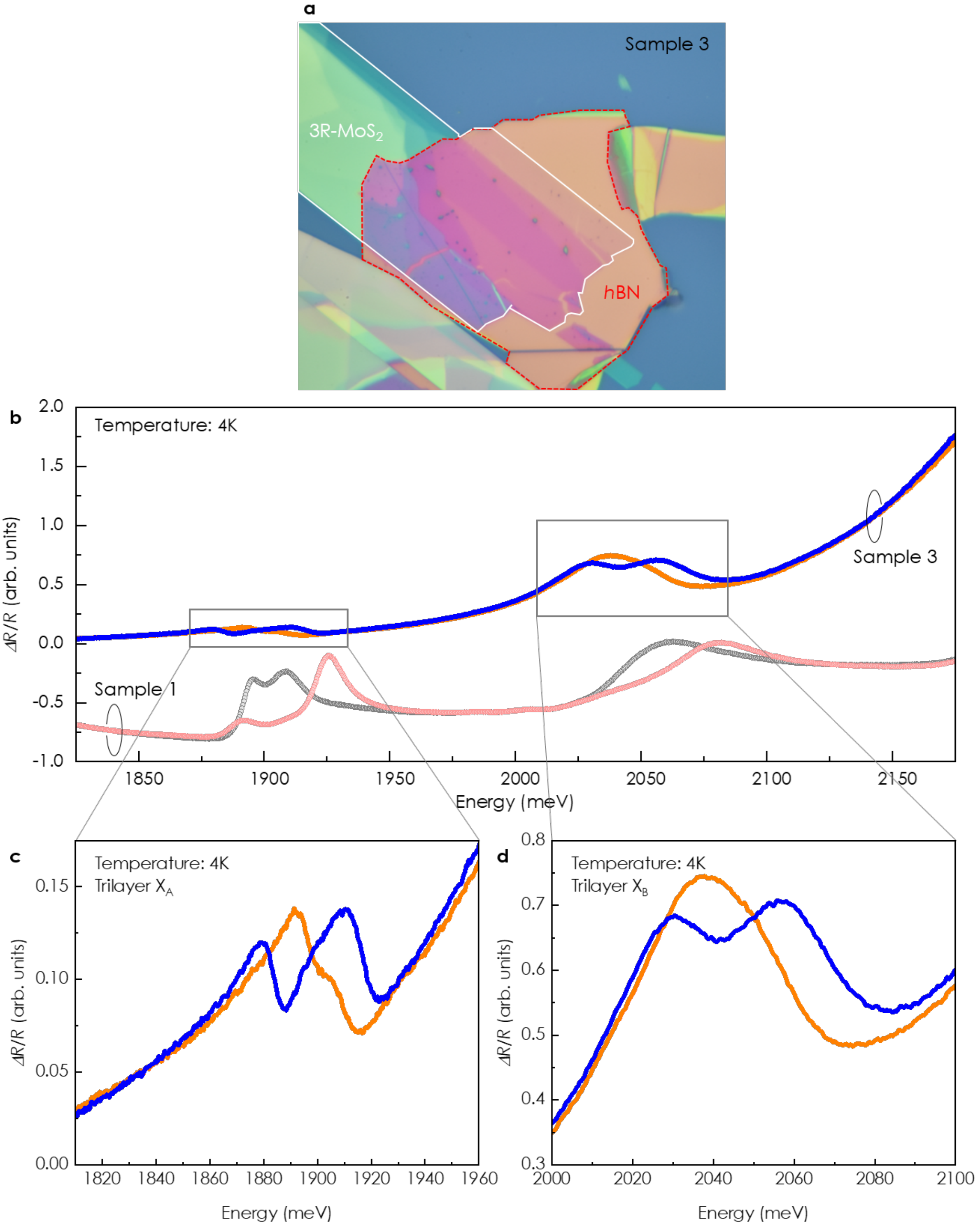

Figure S11 | Cavity effect on reflection contrast intensity. a. Another 3R-MoS_2 sample on a 95 nm-hBN/285 nm-SiO_2/Si substrate. **b-d.** Low-temperature reflectance contrast spectra from various spatial locations from the sample shown in panel a, plotted in solid line. Due to the chosen thickness of hBN and SiO_2 substrate, the reflection contrast at the high energy side increases drastically. The spectra from Fig.1-Sample 1 are included as scatter plots for comparison.

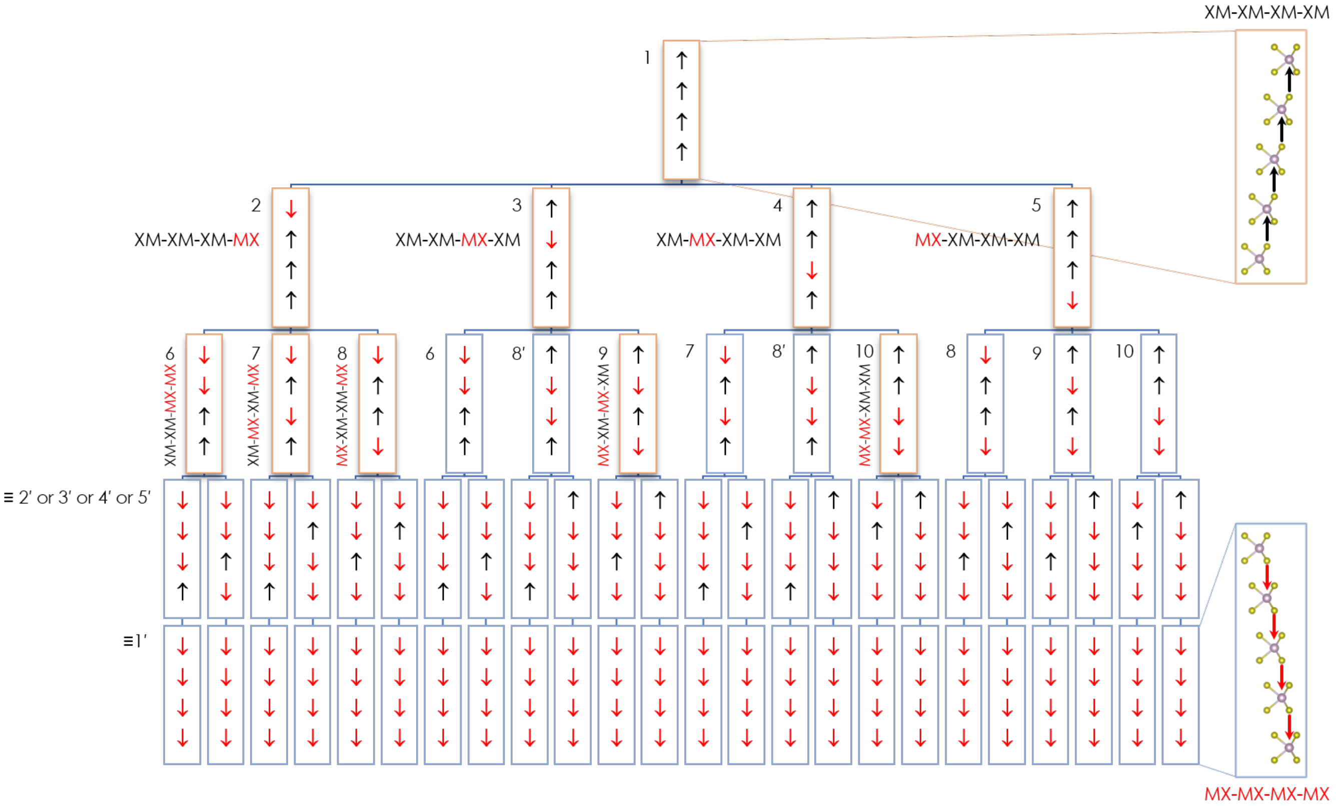

Figure S12 | Possible permutations of stacking order in a five-layer flake. We replace the crystallographic depiction with their respective polarization vector for convenience, for instance, an XM stacking configuration with an upwards black colored arrow and MX as a red downwards arrow. Here, we have taken a systematic approach of depicting all the possible combinations by changing the stacking order of one interface at a time, starting from co-polarized XM-XM-XM-XM (1) configuration to entirely flipped MX-MX-MX-MX (1′) ordering. Orange-colored boxes indicate crystallographically non-equivalent and unique stacking orders. The "′" symbol has been used to indicate equivalent yet flipped configurations. The scheme shows all sixteen possible stacking combinations, out of which ten are crystallographically unique, and the rest are flipped versions of a few. For example, XM-MX-MX-XM (8′) is a 180° flipped version of MX-XM-XM-MX (8). It is interesting to note that KPFM measurement can distinguish among different rows of this schematic representation but is insensitive to the elements within a given row. For example, XM-XM-XM-XM (1) and XM-XM-XM-MX (2) would have different surface potential; however, all the combinations in the second row, from 2 to 5, would remain indistinguishable in a KPFM measurement.

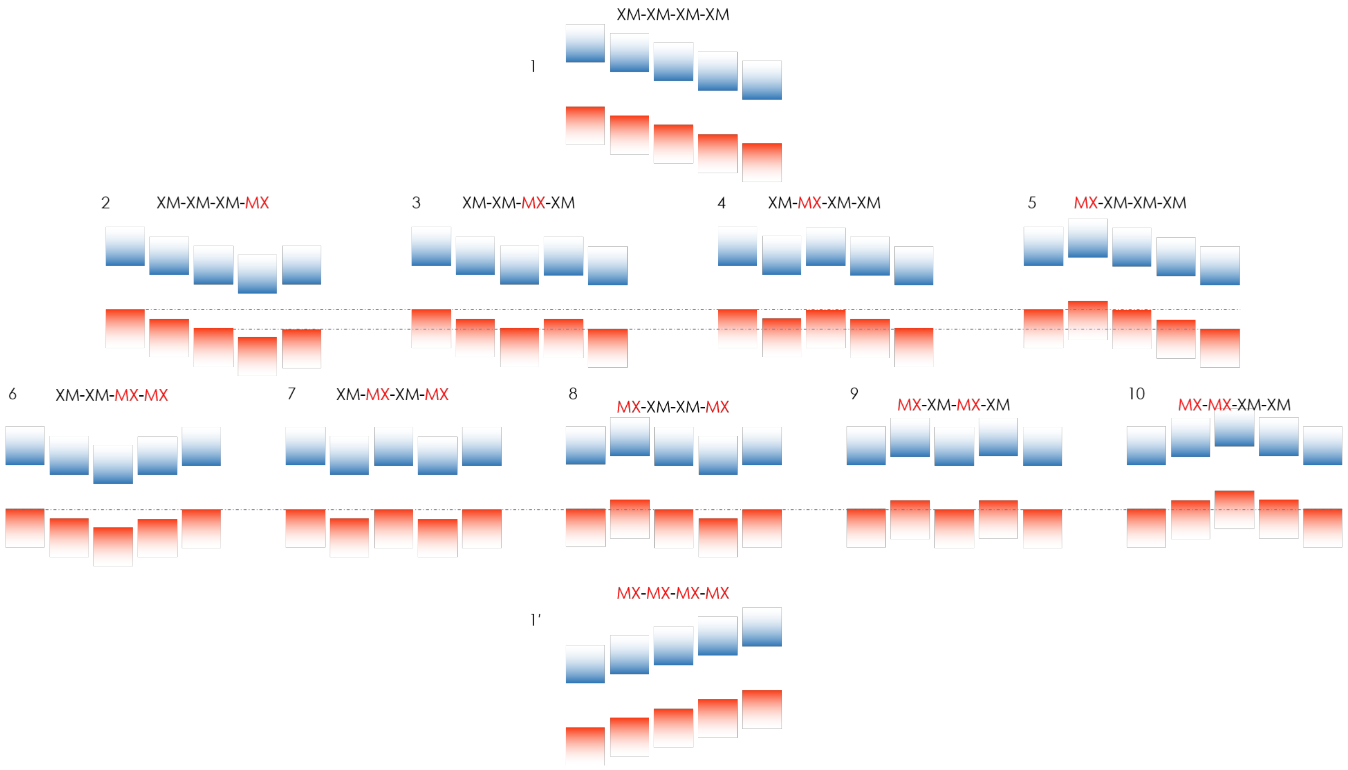

Figure S13 | Schematic of real-space band alignment for different stacking, a purely electrostatic perspective. Schematics of layer-projected K_{VB} and K_{CB} band-edges for crystallographically unique stacking configurations, discussed in Fig. S12. The dashed lines act as a guide to the eye to highlight valence-band degeneracies, which can occur in partially polarized stacking. To first order, the band-edge variation arises due to the rigid shift in layer-projected K valleys introduced by the ferroelectricity-induced electrostatic considerations.

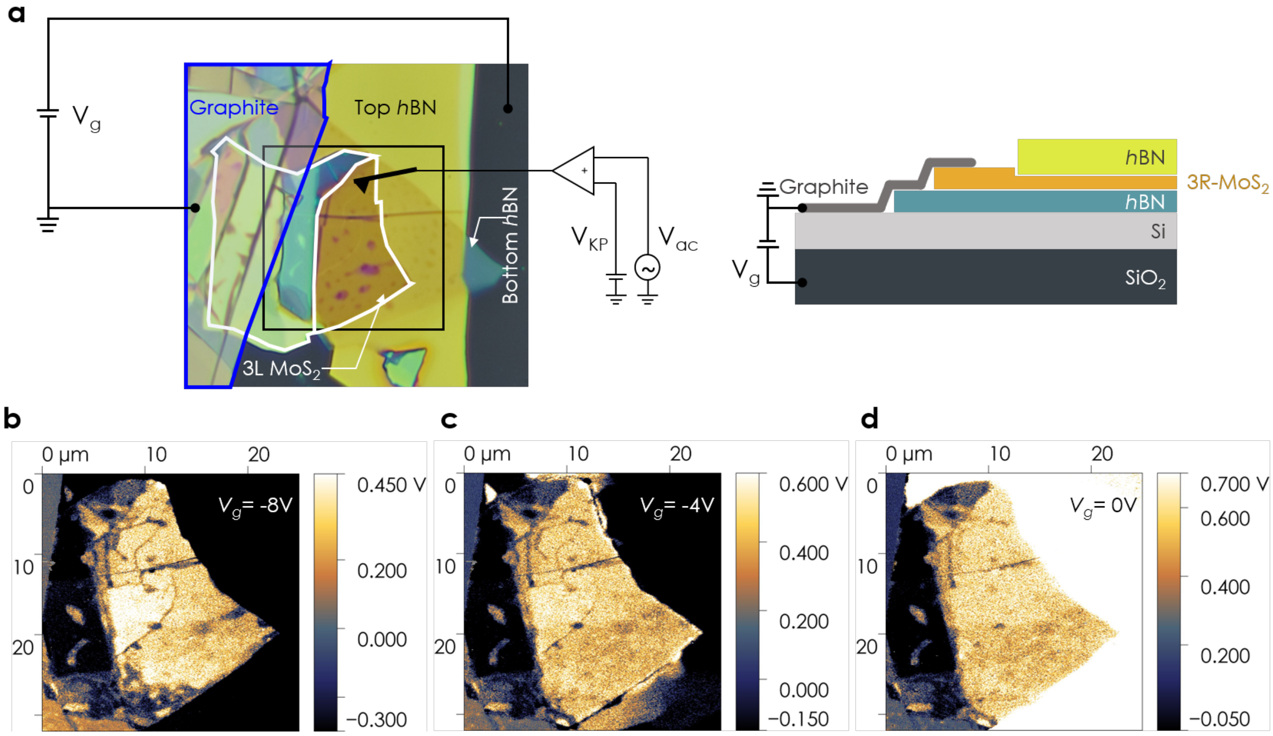

Figure S14 | Characterization of Sample 4. a. Optical micrograph of a top-encapsulated 3L-3R-MoS_2 flake on $Si/SiO_2/h$BN. Topographical steps and edges of the MoS_2 flake have been marked by white lines. We have used blue lines to mark the graphite flake used as a source/drain contact. On the right panel we draw the device schematic from a lateral perspective. **b.** Surface potential map at different gate voltages within the area enclosed by the black rectangle in a. Two domains can be identified by the difference in contrast, which is particularly strong for V_g= -8 V.

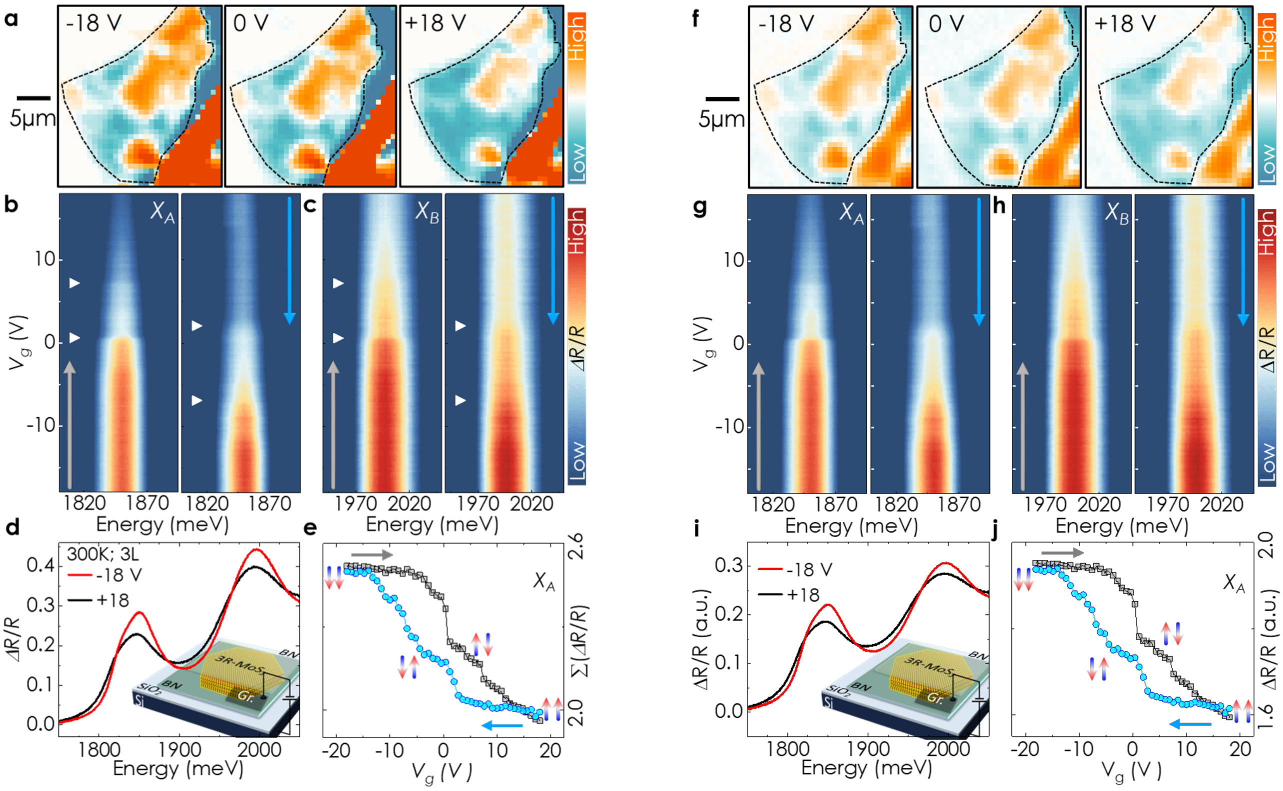

Figure S15 | Comparison of $(\mathbf{R_{Ref}} - \mathbf{R_{Flake}})/\mathbf{R_{Flake}}$ with $(\mathbf{R_{Ref}} - \mathbf{R_{Flake}})/\mathbf{R_{Ref}}$ for Sample 4. a - e Replica of Fig. 3 i.e. RC maps, RC intensity as a function of gate voltage, RC spectra, and its hysteretic response where we have used RC=$(R_{Ref} - R_{Flake})/R_{Flake}$. **(f - j)** the same with RC defined as $(R_{Ref} - R_{Flake})/R_{Ref}$,

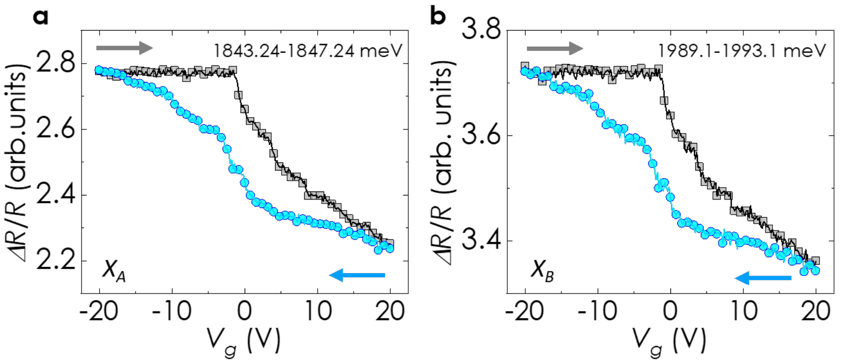

Figure S16 | Integrated RC intensity profile as a function of gate voltage at the A and B excitonic region over an extended range.

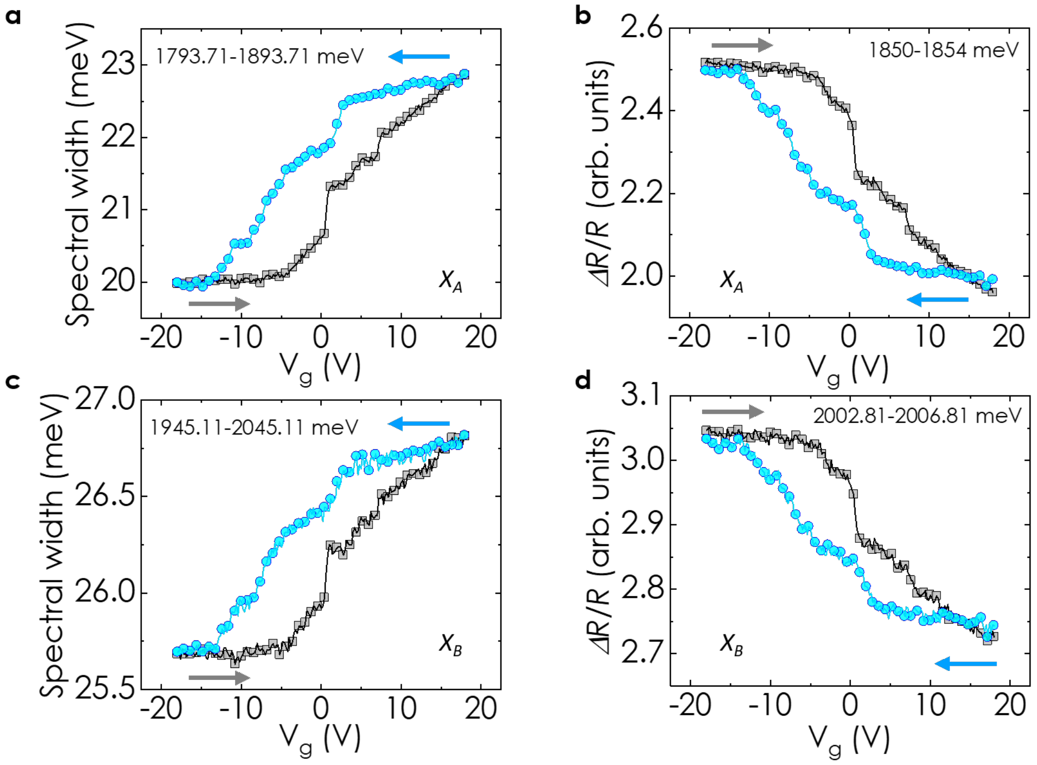

Figure S17 | Integrated RC intensity (≡ line cuts from panel b and c of Fig. 3) and standard deviation profile at the A and B excitonic region. Integrated intensity $= \sum_{x=\mathrm{x_{min}}}^{\mathrm{x_{max}}} \Delta\mathrm{R/R}(x)$; Standard deviation $= \sqrt{\frac{\sum_{x=\mathrm{x_{min}}}^{\mathrm{x_{max}}} \Delta\mathrm{R/R}(x)(x-x_0)^2}{\sum_{x=\mathrm{x_{min}}}^{\mathrm{x_{max}}} \Delta\mathrm{R/R}(x)} - \frac{\sum_{x=\mathrm{x_{min}}}^{\mathrm{x_{max}}} \Delta\mathrm{R/R}(x)(x-x_0)}{\sum_{x=\mathrm{x_{min}}}^{\mathrm{x_{max}}} \Delta\mathrm{R/R}(x)}}$, where $x_0 = (x_{max}+x_{min})/2$, x is the energy of interest. **a.** Standard deviation with $x_{max} = 1893.71meV, x_{min} = 1793.71meV$ as a function of gate voltage. **b.** Integrated intensity with $x_{max} = 1854meV, x_{min} = 1850meV$ as a function of gate voltage (copy of Fig. 3e for completeness). **c.** Standard deviation with $x_{max} = 2045.11meV, x_{min} = 1945.11meV$ as a function of gate voltage. **d.** Integrated intensity with $x_{max} = 2006.81meV, x_{min} = 2002.81meV$ as a function of gate voltage.

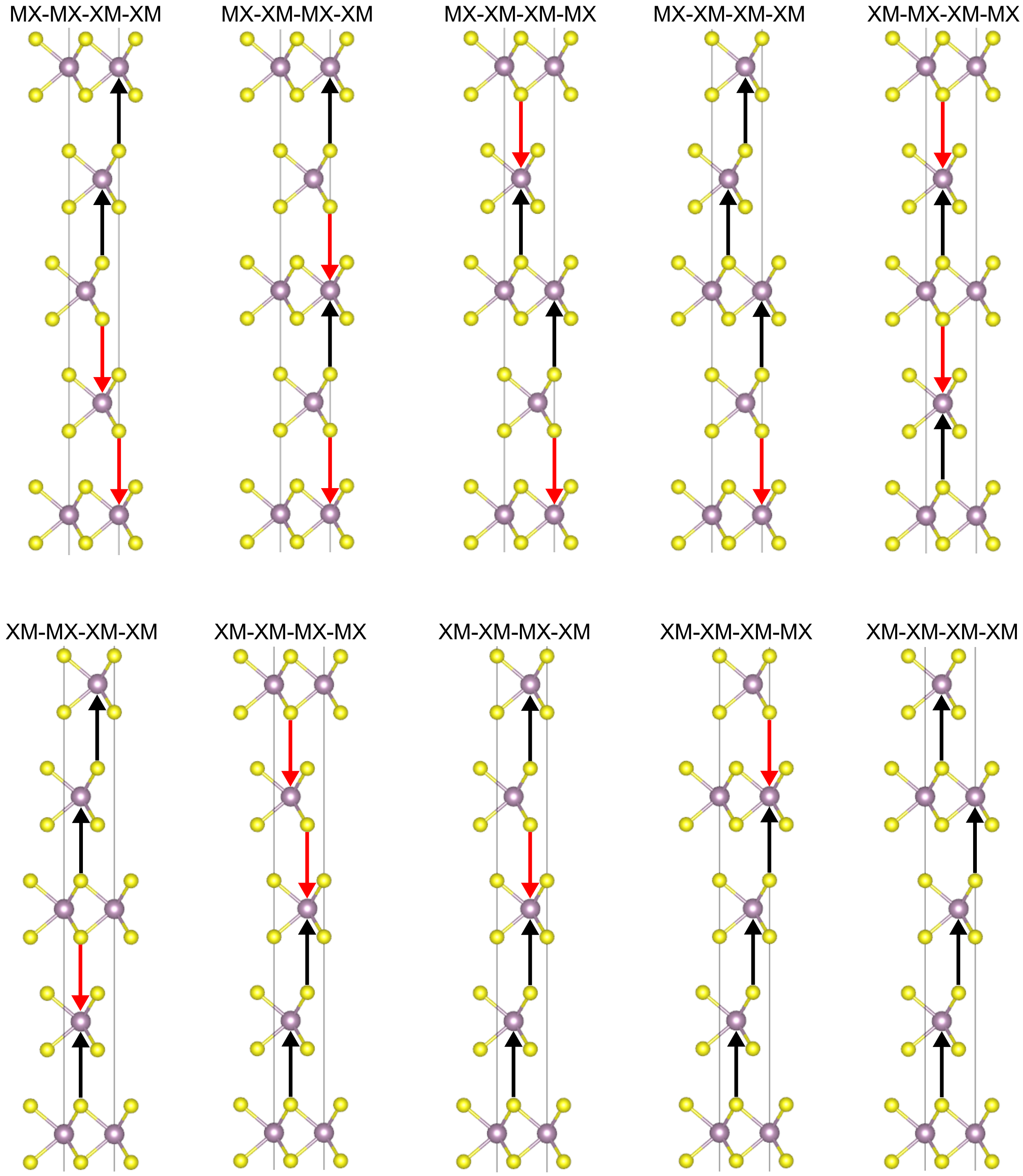

Figure S18 | Crystal structures for the different stackings in 3R 5L MoS_2. Red (black) arrows indicate the MX (XM) label.

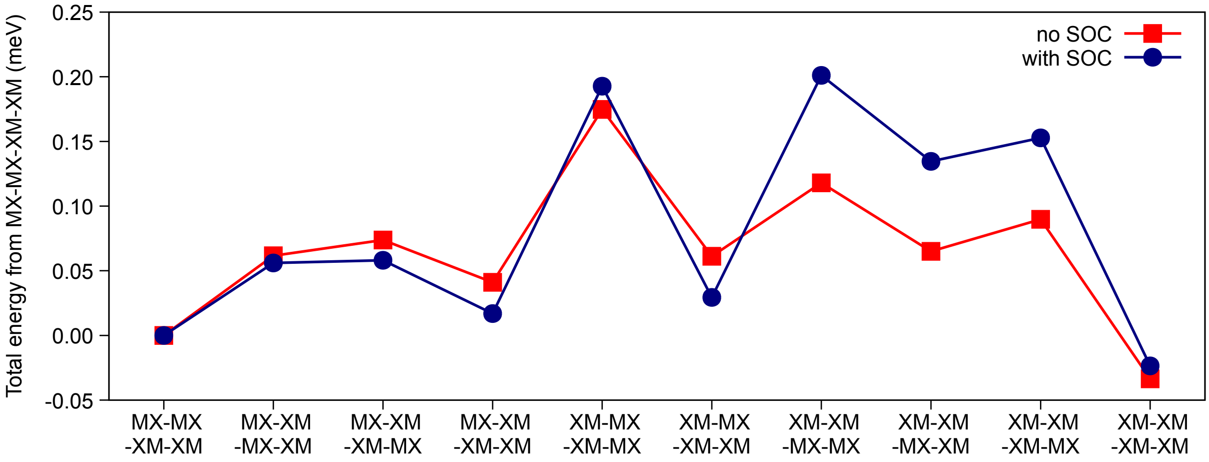

Figure S19 | Total energy (with respect to the MX-MX-XM-XM case) for the different stackings.

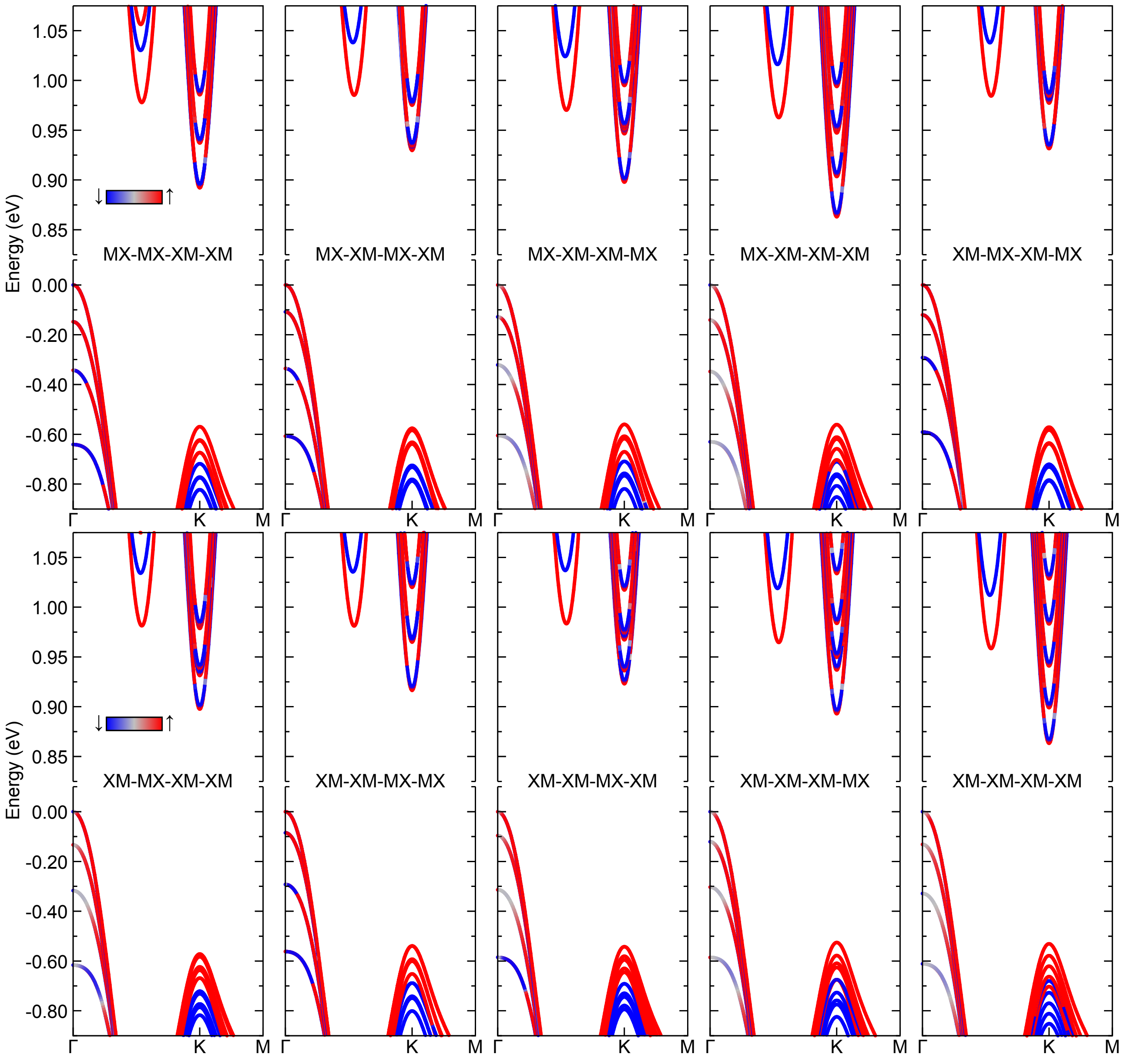

Figure S20 | Band structure along the $\Gamma - K - M$ line, color-coded according to the expectation value of spin along Z, $< s_z >$.

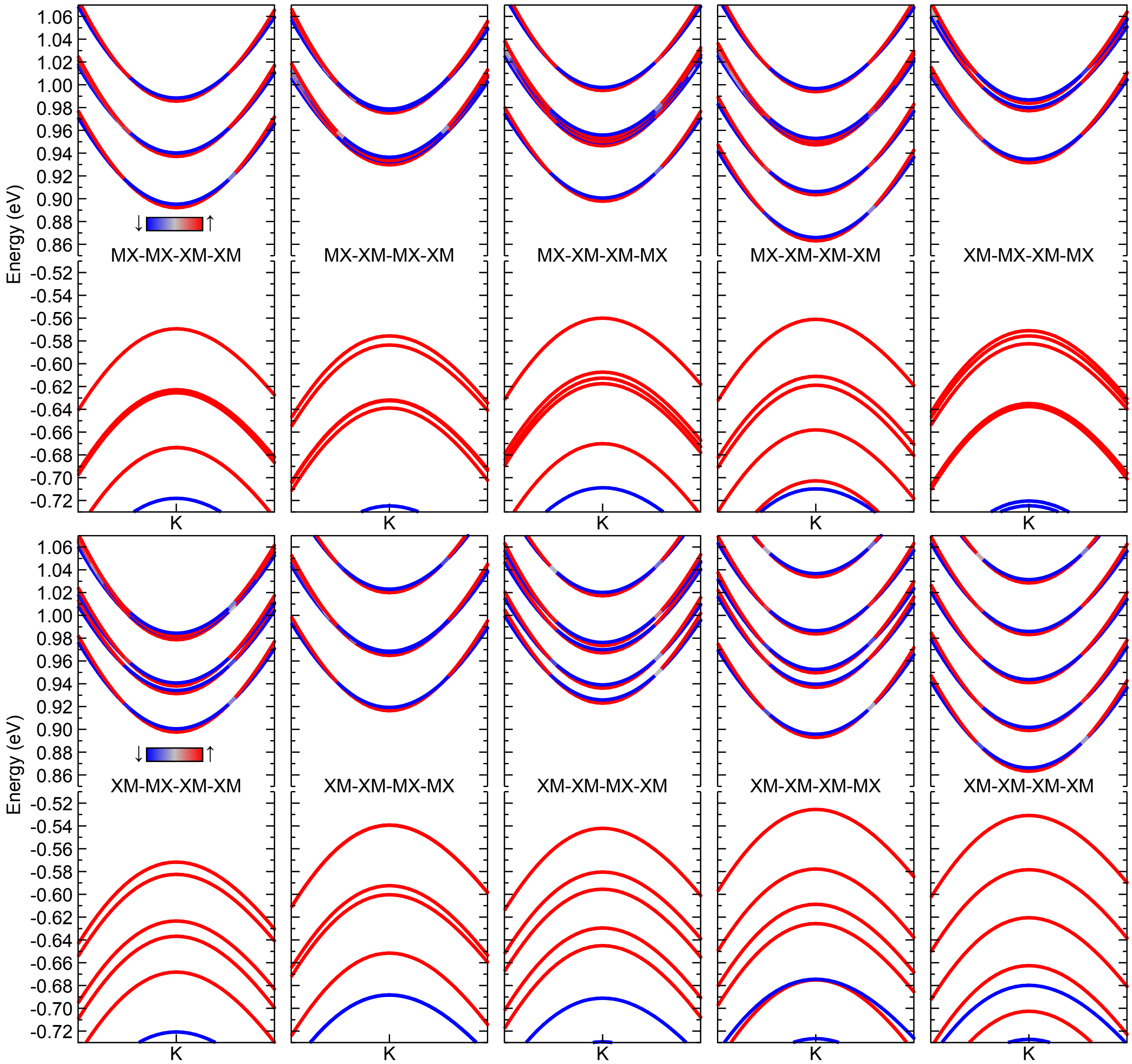

Figure S21 | Band structure with a zoom around the K point.

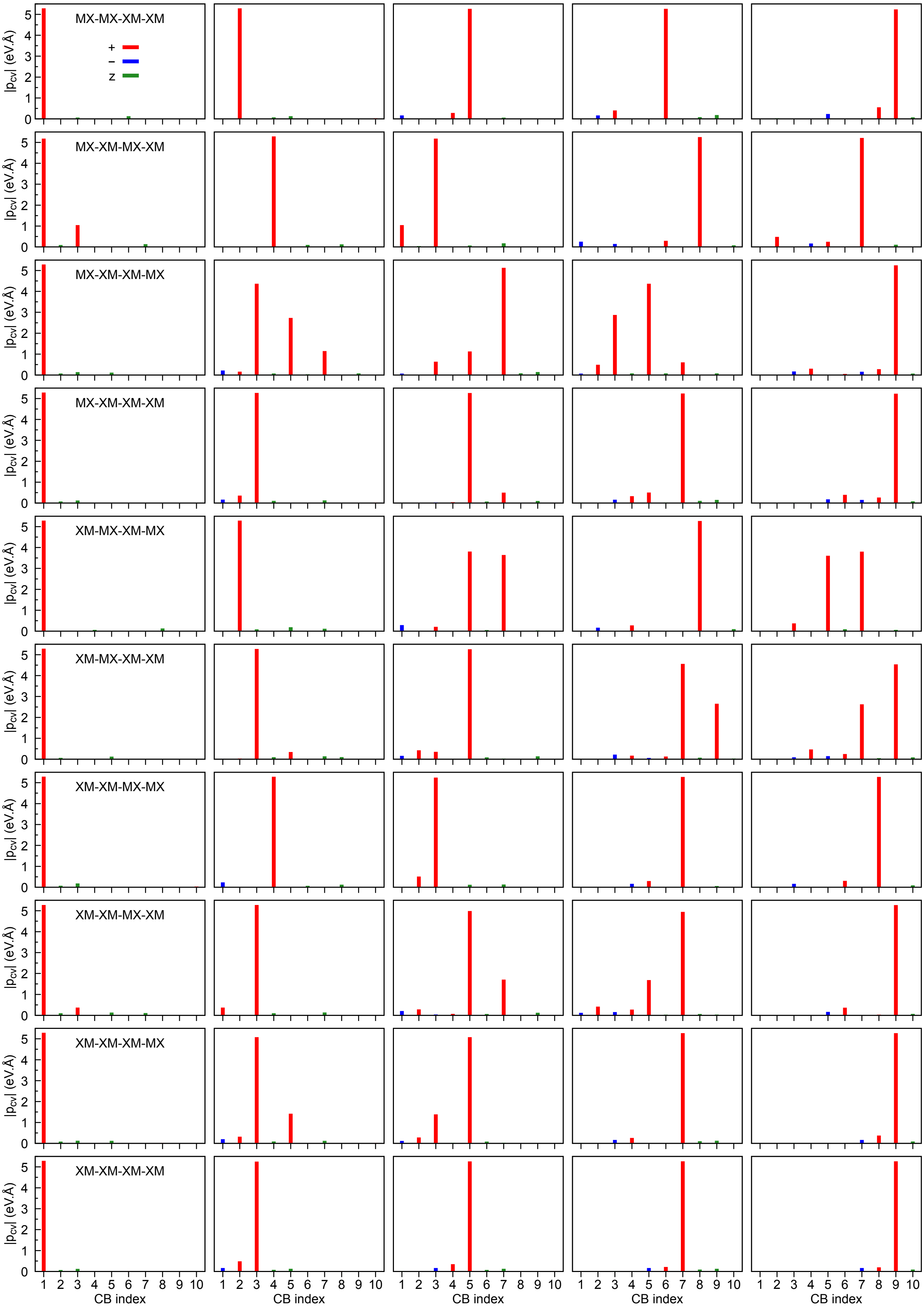

Figure S22 | Absolute value of the dipole matrix elements between the valence bands with spin-up (different columns) to all conduction bands (labeled by index).

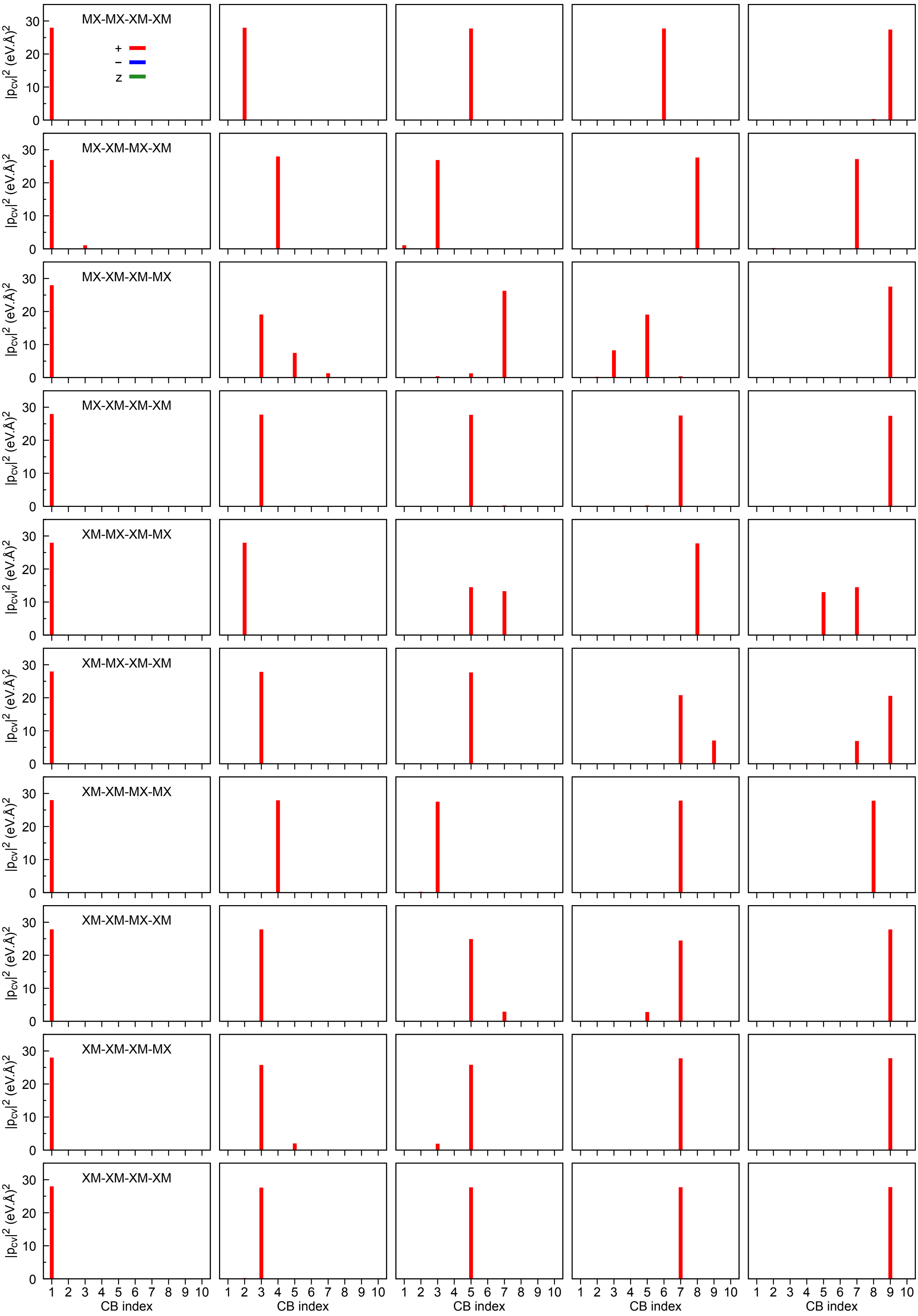

Figure S23 | Same as Fig. S22 but for the oscillator strength, i. e., $|p_{cv}|^2$.

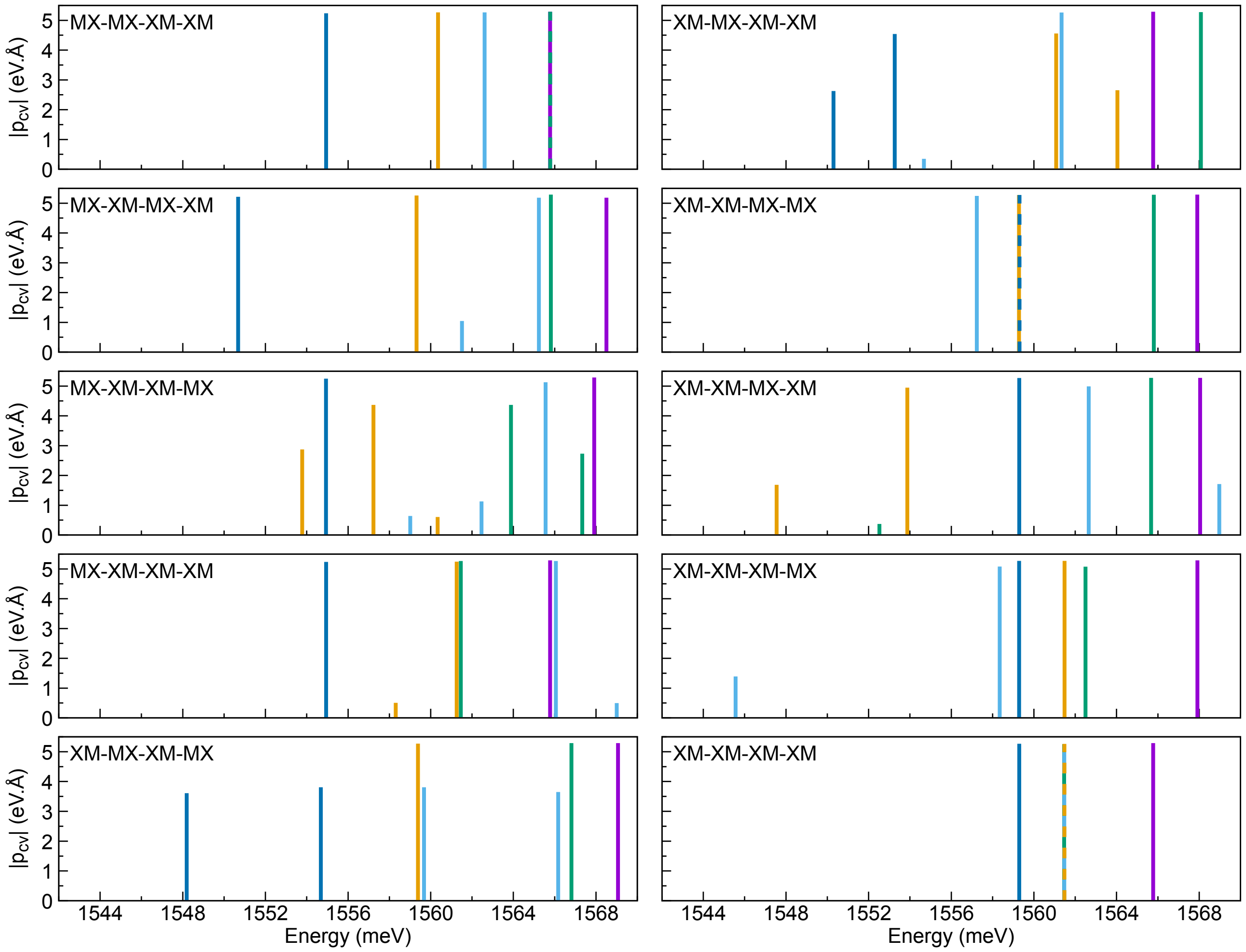

Figure S24 | Absolute value of the dipole matrix elements for s^+ between the valence bands with spin-up and all conduction bands as a function of the transition energy.

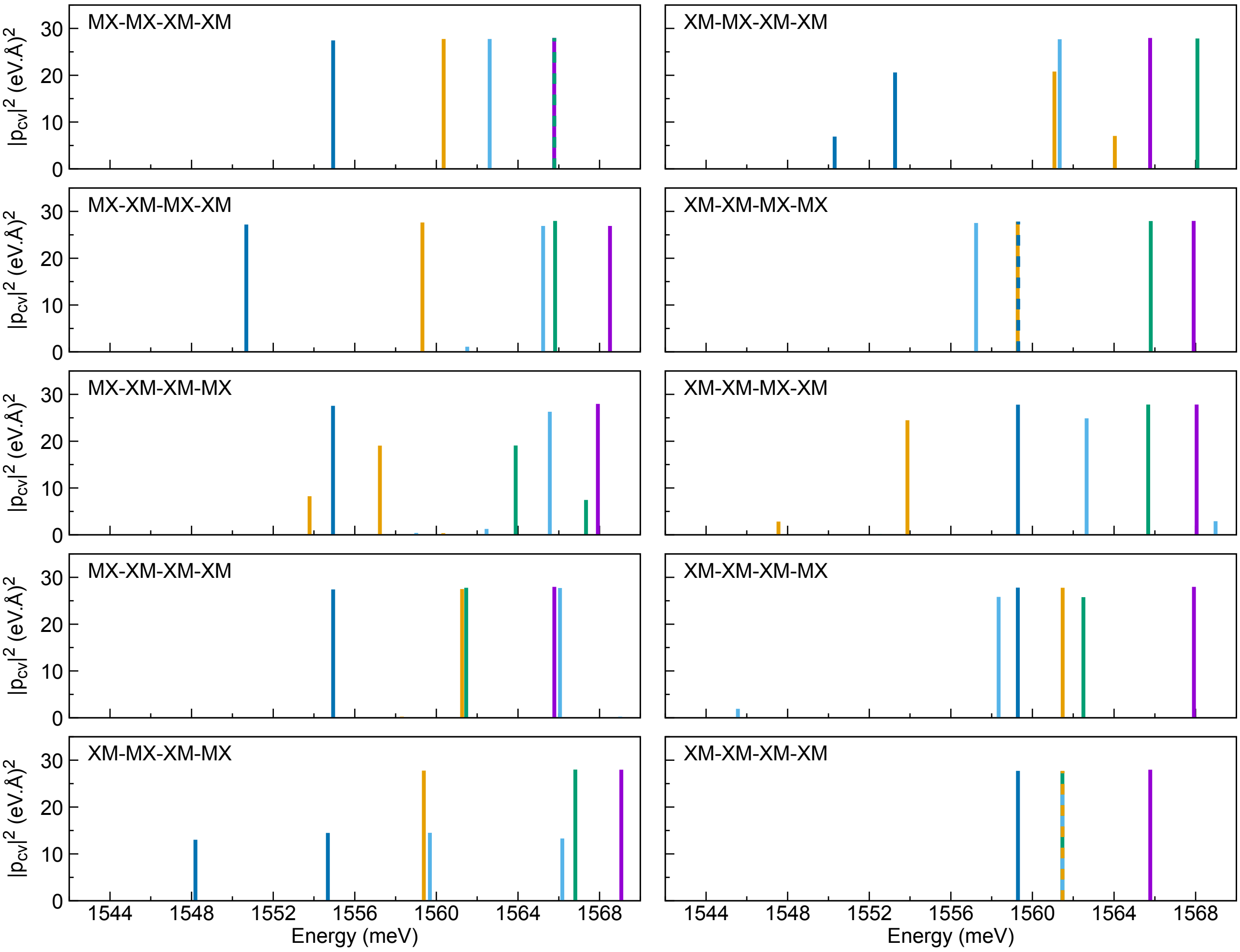

Figure S25 | Same as Fig. S24 but for the oscillator strength, i. e., $|p_{cv}|^2$.

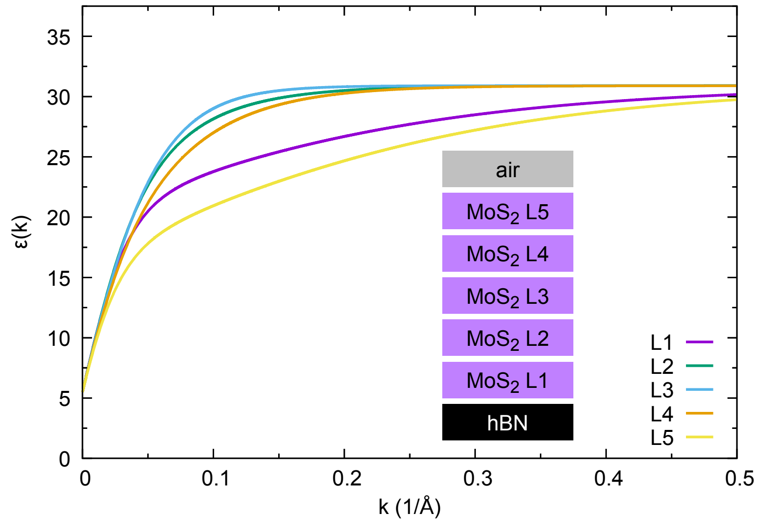

Figure S26 | Layer dependent dielectric constants used in the exciton calculations.

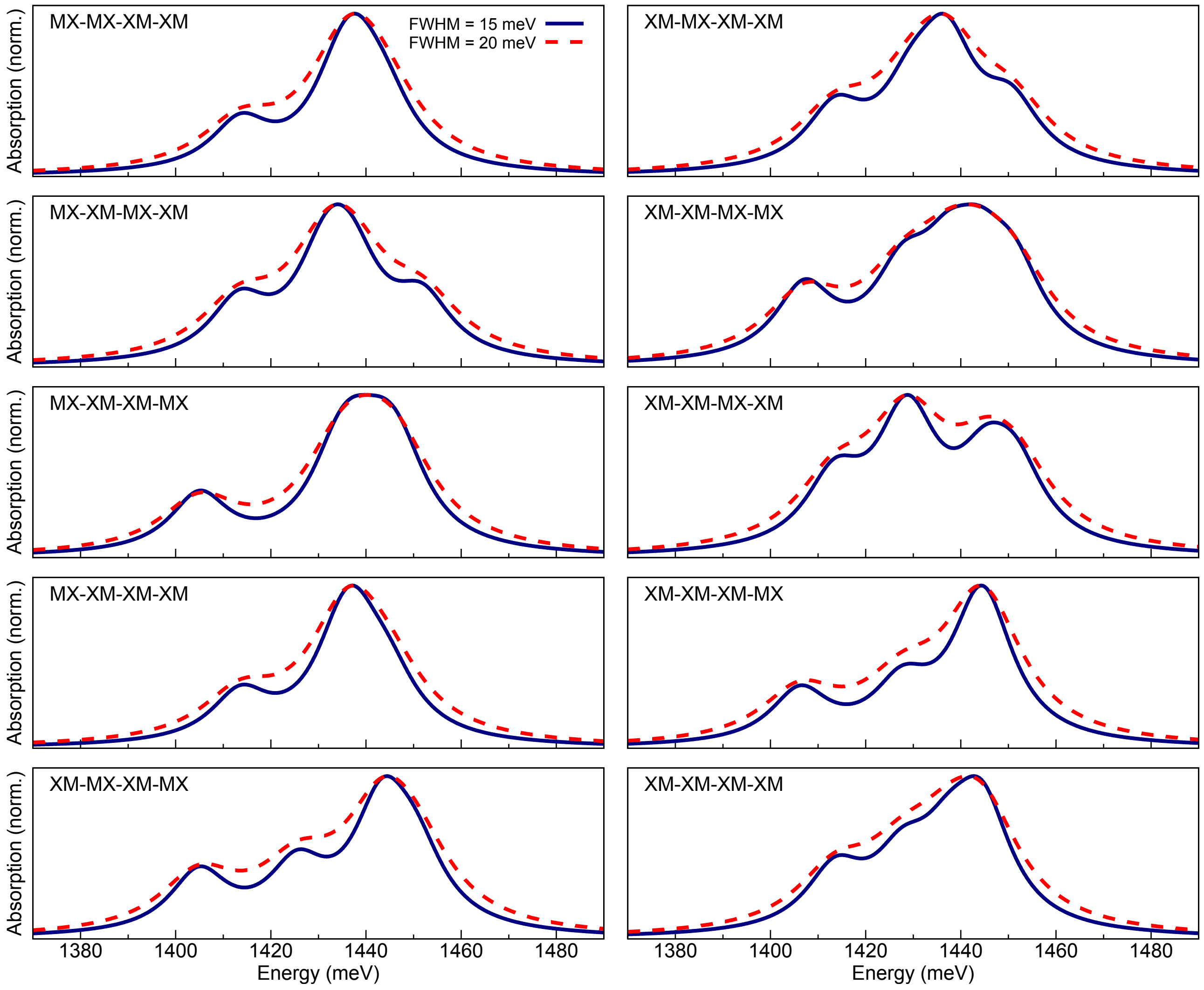

Figure S27 | Exciton absorption spectra for the different stackings using a phenomenological Lorentzian broadening with a full-width-half-maximum of 15 (20) meV shown by solid (dashed) lines.

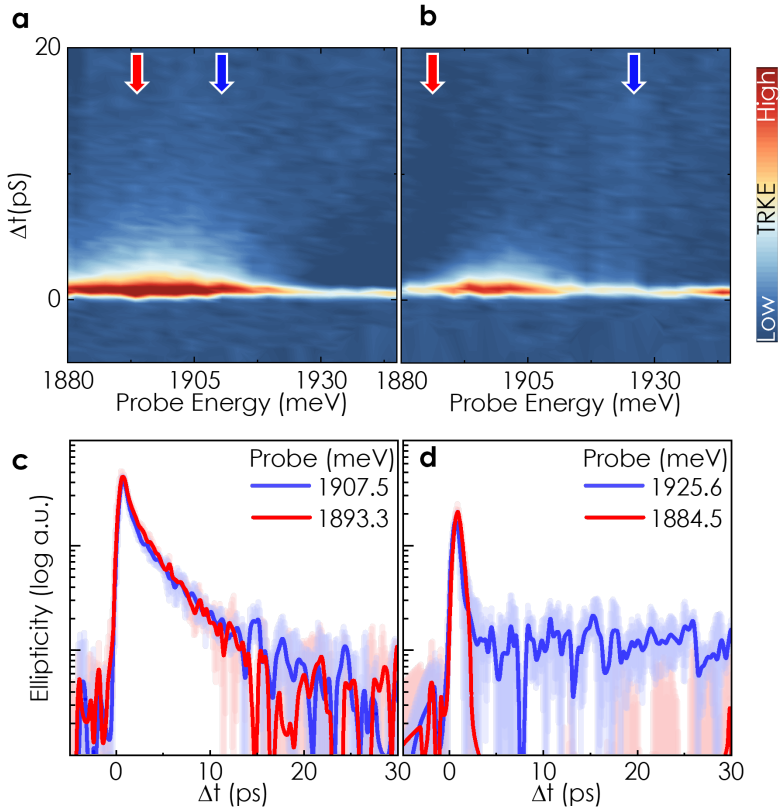

Figure S28 | a-b. False color map of transient ellipticity as a function of probe energy from domain I and II, respectively. **c-d.** Transient reflection at selected energies, marked by colored arrows in the top panels (copied from Fig. 4 of the main text for completeness).

References

[1] Zomer, P., Guimarães, M., Brant, J., Tombros, N. & Van Wees, B. Fast pick up technique for high quality heterostructures of bilayer graphene and hexagonal boron nitride. *Applied Physics Letters* **105** (2014).

[2] Kempf, M., Schubert, A., Schwartz, R. & Korn, T. Two-color kerr microscopy of two-dimensional materials with sub-picosecond time resolution. *Review of Scientific Instruments* **92**, 113904 (2021).

[3] Kempf, M. A. *et al.* Rapid spin depolarization in the layered 2d ruddlesden–popper perovskite (BA)(MA) PbI. *ACS nano* **17**, 25459–25467 (2023).

[4] Blaha, P. *et al.* Wien2k: An apw+lo program for calculating the properties of solids. *The Journal of Chemical Physics* **152**, 074101 (2020).

[5] Perdew, J. P., Burke, K. & Ernzerhof, M. Generalized gradient approximation made simple. *Physical Review Letters* **77**, 3865 (1996).

[6] Grimme, S., Antony, J., Ehrlich, S. & Krieg, H. A consistent and accurate ab initio parametrization of density functional dispersion correction (DFT-D) for the 94 elements H-Pu. *The Journal of Chemical Physics* **132**, 154104 (2010).

[7] Singh, D. J. & Nordstrom, L. *Planewaves, Pseudopotentials, and the LAPW method* (Springer Science & Business Media, 2006).

[8] Kim, H.-g. & Choi, H. J. Thickness dependence of work function, ionization energy, and electron affinity of mo and w dichalcogenides from dft and gw calculations. *Phys. Rev. B* **103**, 085404 (2021).

[9] Ambrosch-Draxl, C. & Sofo, J. O. Linear optical properties of solids within the full-potential linearized augmented planewave method. *Computer Physics Communications* **175**, 1–14 (2006).

[10] Rohlfing, M. & Louie, S. G. Electron-hole excitations in semiconductors and insulators. *Physical Review Letters* **81**, 2312 (1998).

[11] Rohlfing, M. & Louie, S. G. Electron-hole excitations and optical spectra from first principles. *Physical Review B* **62**, 4927 (2000).

[12] Ovesen, S. *et al.* Interlayer exciton dynamics in van der waals heterostructures. *Communications Physics* **2** (2019).

[13] Faria Junior, P. E. *et al.* Proximity-enhanced valley zeeman splitting at the WS_2/graphene interface. *2D Materials* **10**, 034002 (2023).

[14] Laturia, A., Van de Put, M. L. & Vandenberghe, W. G. Dielectric properties of hexagonal boron nitride and transition metal dichalcogenides: from monolayer to bulk. *npj 2D Materials and Applications* **2**, 6 (2018).

[15] Stier, A. V. *et al.* Magnetooptics of exciton rydberg states in a monolayer semiconductor. *Phys. Rev. Lett.* **120**, 057405 (2018).

[16] Berkelbach, T. C., Hybertsen, M. S. & Reichman, D. R. Theory of neutral and charged excitons in monolayer transition metal dichalcogenides. *Phys. Rev. B* **88**, 045318 (2013).

[17] Deb, S. *et al.* Cumulative polarization in conductive interfacial ferroelectrics. *Nature* **612**, 465–469 (2022).

[18] Cao, W. *et al.* Polarization saturation in multi-layered interfacial ferroelectrics. *Advanced Materials* 2400750 (2024).
